# Cytokine profile and nitric oxide levels in peritoneal macrophages of BALB/c mice exposed to the fucose-mannose ligand of *Leishmania infantum* combined with glycyrrhizin

**DOI:** 10.1186/s13071-020-04243-7

**Published:** 2020-07-20

**Authors:** Hasan Namdar Ahmadabad, Reza Shafiei, Gholam Reza Hatam, Reza Zolfaghari Emameh, Ashok Aspatwar

**Affiliations:** 1 Natural Products & Medicinal Plants Research Center, North Khorasan University of Medical Sciences, Bojnurd, Iran; 2 Vector-borne Diseases Research Center, North Khorasan University of Medical Sciences, Bojnurd, Iran; 3 Department of Parasitology and Mycology, Shiraz University of Medical Sciences, Shiraz, Iran; 4 Department of Energy and Environmental Biotechnology, National Institute of Genetic Engineering and Biotechnology (NIGEB), 14965/161, Tehran, Iran; 5 Faculty of Medicine and Health Technology, Tampere University, 33014 Tampere, Finland

**Keywords:** Fucose-mannose ligand, Glycyrrhizin, Macrophage, Nitric oxide, Visceral leishmaniasis

## Abstract

**Background:**

The fucose-mannose ligand (FML) of *Leishmania infantum* is a complex glycoprotein which does not elicit adequate immunogenicity in humans. In recent years, adjuvant compounds derived from plants have been used for improving the immunogenicity of vaccines. Glycyrrhizin (GL) is a natural triterpenoid saponin that has known immunomodulatory activities. In the present study, we investigated the effects of co-treatment with FML and GL on the production of cytokines and nitric oxide **(**NO) by macrophages, *in vitro*.

**Methods:**

Lipopolysaccharide (LPS) stimulated murine peritoneal macrophages were treated with FML (5 μg/ml) of *L. infantum* and various concentrations of GL (1 μg/ml, 10 μg/ml and 20 μg/ml). After 48 h of treatment, cell culture supernatants were recovered and the levels of TNF-α, IL-10, IL-12p70 and IP-10 were measured by sandwich ELISA and NO concentration by Griess reaction.

**Results:**

Our results indicate that the treatment of activated macrophages with FML plus GL leads to enhanced production of NO, TNF-α and IL-12p70, and reduction of IL-10 levels in comparison with FML treatment alone.

**Conclusions:**

Therefore, we concluded that GL can improve the immunostimulatory effect of FML on macrophages and leads to their polarization towards an M1-like phenotype. 
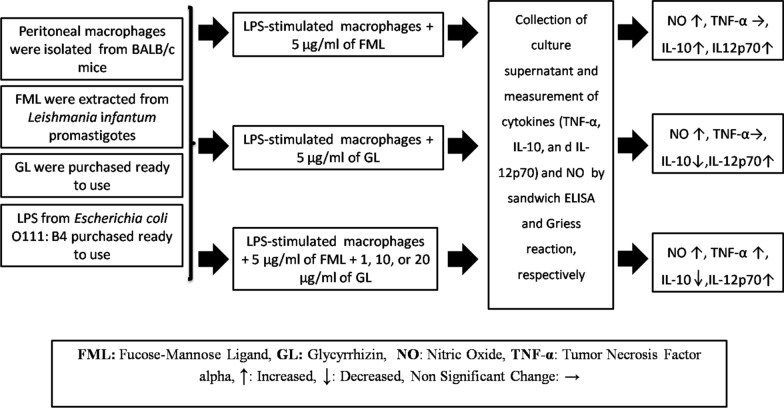

## Background

Visceral leishmaniasis (VL) or kala-azar, a cosmopolitan vector-borne zoonotic disease, is the most severe and fatal form of leishmaniasis if not diagnosed and treated in time. The disease is caused by parasitic protozoan species of the *Leishmania donovani* complex and is transmitted by sand flies [1]. It is one of the most important parasitic diseases among the many parasitic diseases in Iran [2–5].

In the recent past, the parasites have developed resistance to existing drugs; due to this and the lack of an effective human vaccine against VL, there has been an increase in the incidence of VL [6–9]. Different generations of vaccines against the different regions of parasitic antigens have been examined in the hope of finding an appropriate treatment for leishmaniasis or induce protection against the disease with long-term immunity [9–12].

Among the various antigens that serve as targets for VL vaccine design, fucose-mannose ligand (FML) has attracted much attention owing to its excellent immunoprotective properties against experimental VL in several animal models [13]. The FML is a glycoprotein antigen which is present both in amastigotes and in motile promastigotes of species in the *L. donovani* complex. Even though the FML is a potent immunogen in rabbits and dogs (e.g. Leishmune®, a vaccine for canine VL consisting of FML and saponin) [14], it does not have adequate immunogenicity in humans [9, 15].

Previous studies have demonstrated that macrophages play a pivotal role in the outcome of *Leishmania* infection depending on the type of macrophages: classically activated (M1) macrophages as efficient type against *Leishmania* parasites, or alternatively, activated (M2) macrophages as favoring survival and growth of *Leishmania* parasites [16, 17]. In response to different microbial stimuli and immune status of the microenvironment, naive macrophages (M0) differentiate into either M1 or M2 subpopulations with different patterns of cytokine production and distinct properties. Concerning the stimuli that can affect shifting of macrophages towards M1 or M2 subpopulations, recently we evaluated the immunomodulatory effects of FML on macrophages [18]. Our findings showed that although the FML significantly increases nitric oxide (NO), IL-12p70 and IP-10 production in the macrophages, but cannot alter TNF-α production in them [18]. The most surprising aspect of this study was that FML significantly increased the production of IL-10, an immunosuppressive cytokine, from macrophages [18].

Glycyrrhizin (GL) is a natural triterpenoid saponin derived from the root of licorice (*Glycyrrhiza glabra*) that has been associated with numerous pharmacologic effects, including anti-bacterial, anti-inflammatory, anti-oxidant, anti-viral, anti-tumor, hepatoprotective, and immunomodulatory activities [19–22]. Stimulation of IL-12 and NO production and suppression of IL-10 production from macrophages [23, 24], augmentation of NK cell activity [23], upregulation of costimulatory molecules on dendritic cells [19], increase in T cell proliferation, reduction of IL-4 production from T cells, and direction of the immune response towards Th1 [19], are the most important known immunomodulatory activities of GL.

It is well known that the GL plays an important role in immunomodulatory activities such as stimulation of IL-12 and NO production, and suppression of IL-10 production from macrophages [23, 24]. GL also plays a central role in the augmentation of NK cell activity, upregulation of costimulatory molecules on dendritic cells, increasing T cell proliferation, reduction of IL-4 production from T cells, and direction of the immune response towards Th1 [19].

With regard to improvements in the production of a vaccine against human VL by using purified FML, and considering the importance of macrophages in protection and control against VL, it seems that characterization of the immunomodulatory effects of a combination of FML/GL on macrophages can be useful in finding a way to increase the immunogenicity of FML and vaccine development. Therefore, to the best of our knowledge, for the first time, we investigated the effects of FML/GL on production of cytokines and NO by macrophages, *in vitro*.

With regard to the improvement in producing human VL vaccines by using purified FML, and considering the importance of macrophages in the protection and control of VL, it is important to characterize the immunomodulatory effects of a combination of FML/GL on macrophages which can be useful in finding ways to increase the immunogenicity of FML and vaccine development.

## Methods

### Animals

For the experimental studies, a total of seven 6–8 week-old female BALB/c mice were used from Razi Institute (Mashhad, Iran). The mice were fed standard mouse chow *ad libitum* throughout the study and maintained under the standard conditions according to the protocol.

### *Leishmania* promastigote culture and FML extraction

*Leishmania infantum* promastigotes (MCAN/IR/07/Moheb-gh) were grown at 26 °C in brain heart infusion broth (37 g/l; Himedia, Mumbai, India) supplemented with 10% of fetal bovine serum (Gibco, Paisley, UK), hemin (0.01 g/l) and folic acid (0.02 g/l; Sigma-Aldrich, St. Louis, MO, USA).

The stationary phase growth medium was centrifuged at 6000× *g* for 10 min to collect the promastigotes. The pellet containing promastigotes was washed with cold phosphate-buffered saline (PBS) and was stored at − 20 °C until further analysis. Aqueous extraction of FML was carried out as previously described by Foroughi-Parvar et al. [25]. Briefly, frozen pellets of the parasite were mixed with cold distilled water and centrifuged at 6000×*g* for 10 min to collect the supernatant. This step was repeated once again, and both the supernatants were combined and boiled for 15 min at 100 °C. The sample was then centrifuged at 6000 for 20 min, and the supernatant was lyophilized and subjected to chromatography by loading 2 ml of lyophilized sample in cold deionized distilled water (10 mg/ml) on 100 × 1.6 cm column of P10 Bio-Gel (Bio-Rad, Watford, UK) to purify the FML. The collected FML samples were analyzed further for their carbohydrate content and for the presence of 10–96 kDa bands corresponding to FML glycoprotein on 10% SDS-PAGE. The purified FML samples were lyophilized and stored at − 20 °C until further use.

### Isolation of peritoneal macrophages

Seven female BALB/c mice were sacrificed by CO_2_ euthanasia. Isolation of peritoneal macrophages was performed based on the procedure described by Bibak et al [26]. Briefly, murine peritoneal cells were harvested by lavage of the peritoneal cavity with 10 ml of RPMI 1640 medium (Invitrogen, Darmstadt, Germany). The cells were centrifuged at 200×*g* for 10 min and washed in PBS/cold ddH_2_O. The cells were then cultured in RPMI 1640 medium in petri dishes at 37 °C for 4 h. In this experiment, we considered three control groups: (i) PBS group: activated macrophages were cultured in the presence of PBS alone at the same volume as the other additions (10 μl/ml); (ii) GL group: activated macrophages treated with GL (5 μg/ml); and (iii) FML group: activated macrophages treated with FML (5 μg/ml). Petri dishes were carefully washed using Hanks’ solution to remove the non-adherent cells. The cells adhered to the Petri dishes were trypsinized and the cell concentration was adjusted to 1 × 10^6^ cells/ml in RPMI medium (RPMI medium with 10% FCS (Invitrogen) containing 50 IU/ml penicillin/streptomycin (Sigma-Aldrich).

### Treatment of peritoneal macrophages with a combination of FML and GL

Isolated peritoneal macrophages were stimulated with 10 μg/ml of LPS at 37 °C and 5% CO_2_ for 4 h. The activated macrophages were treated with FML together with varying concentrations of GL to assess the immunomodulatory effects of the combination of FML and GL. To prepare activated macrophages, 2 × 10^5^ macrophage cell suspensions (200 μl/well in 96-well flat-bottom plates) in each well were treated with 10 μg/ml of LPS from *Escherichia coli* O111: B4 (Sigma-Aldrich) in complete RPMI 1640 medium. Thereafter, 5 μg/ml of FML and 1, 10 and 20 μg/ml of GL (Sigma-Aldrich) mixtures were added to the activate cells in the wells in triplicate, as previously described [25, 27]. The cells were then cultured at 37 °C and 5% CO_2_ for 48 h. In the control or PBS group, activated macrophages were cultured in the presence of PBS alone using the same volume as the other additions (10 μl/ml). For the NO assay, we used only complete RPMI 1640 medium as the blank control group. After 48 h, culture supernatants from each well of the 96-well plate were collected and stored at -80 °C until further analysis. Each experiment was performed in triplicate. The different study groups are shown in Table 1. The MTT (3-(4,5-dimethylthiazol-2-yl)-2,5-diphenyltetrazolium bromide (Merck, Darmstadt, Germany) assay was performed to evaluate the macrophage viability after 48 h of incubation at 37 °C with different concentrations of GL (0.1, 1, 10, 20, 50 and 100 μg/ml).

**Table 1:** Treatment of peritoneal macrophages with GL, FML and a combination of FML and different concentrations of GL in the presence of LPS

Study group no.	1	2	3	4	5	6
LPS (10 μg/ml)	+	+	+	+	+	+
PBS^a^	+	–	–	–	–	–
GL	–	*+* (5 μg/ml)	*–*	*+* (1 μg/ml)	*+* (10 μg/ml)	*+* (20 μg/ml)
FML (5 μg/ml)	–	–	+	+	+	+

^a^Phosphate-buffered saline

### Nitric oxide and cytokine assay

The culture supernatants were evaluated for stable end-products of NO, nitrates, and nitrites, using Standard Griess Reagent according to the instructions provided in the manual (Cayman Chemical, Michigan, USA). Levels of NO in different treatment groups were determined by reading the absorbances at 540 nm in a microplate reader (BioTek, Winooski, Vermont, USA). The mean optical density (OD) values of the blank were subtracted from the mean OD values of the test groups. The concentration of the nitrite was calculated from the standard curve obtained with serial dilutions of sodium nitrite as the standard. Presence of TNF-α, IL-10 and IL-12p70 were measured in the cell culture supernatants using sandwich ELISA kits according to the instructions of the manufacturer (eBioscience, San Diego, CA, USA). The minimum detectable concentration was 5 pg/ml for both IL-12p70 and IL-10, and 1 pg/ml for TNF-α.

### Statistical analysis

GraphPad Prism software version 5.0 (GraphPad Software, San Diego, USA) was used for statistical analysis of the data. Data distribution was analyzed by a Kolmogorov-Smirnov test. According to the results of the normality test, a one-way ANOVA followed by Dunn’s or Tukey’s *post-hoc* test or a non-parametric Kruskal-Wallis test were used for statistical comparisons. Data are shown as the mean ± SD of three independent experiments. *P*-values < 0.05 were considered statistically significant.

## Results

After the treatment of LPS stimulated macrophages with FML and different concentrations of GL, we determined the viability of cells using an MTT reduction assay. Similarly, we measured the levels of TNF-α, IL-10 and IL-12p70 using a sandwich ELISA method and NO concentration using a Griess reaction.

**Fig. 1:**
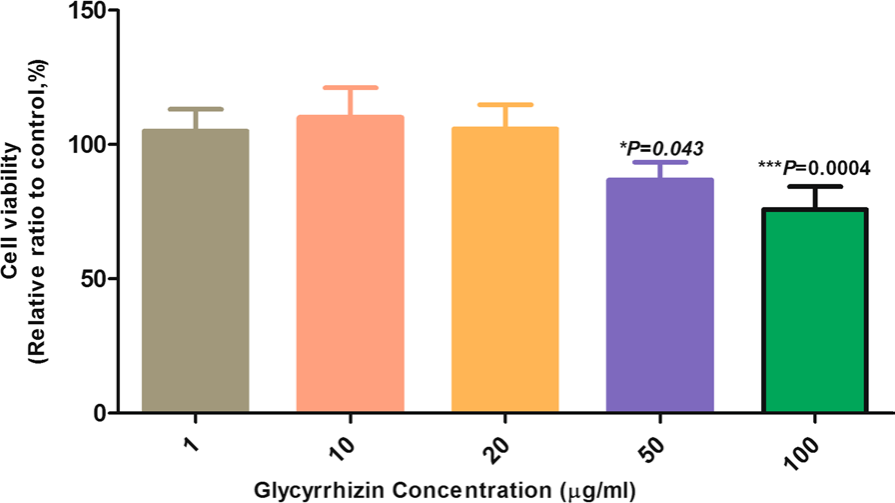
Peritoneal macrophage viability in the presence of different concentrations of glycyrrhizin (GL). The average values of MTT reduction in activated macrophages treated with different concentrations of GL were the same in all the groups except at concentrations of 50 and 100 μg/ml (*P < 0.05, ***P < 0.0001)

Results of the MTT assay indicated that co-treatment of macrophages with FML (5 μg/ml) and GL (1, 10 and 20 μg/ml) had no cytotoxic effect on the activated macrophages, thus these concentrations were used for the NO and cytokine assay. Since the activated macrophages treated with GL at 50 and 100 μg/ml exhibited low viability (< 90%), they were not included in further analysis (Fig. 1).

**Fig. 2:**
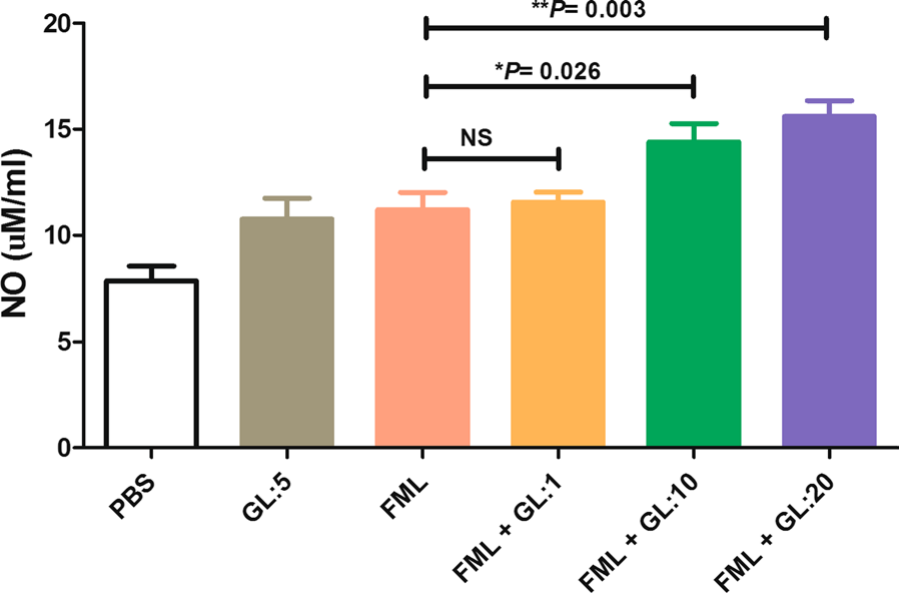
The effect of the combination of fucose-mannose ligand (FML) with glycyrrhizin (GL) on the production of nitric oxide (NO) by activated macrophages. Co-treatment with FML and GL significantly increased NO production in activated macrophages in comparison with activated macrophages treated with PBS, FML or GL alone. Results represent the mean (mean ± SD) of three independent experiments with macrophages from seven mice per experiment (*P < 0.05, **P < 0.01). *Abbreviations*: PBS, phosphate-buffered saline; ns, not significant

The concentrations of NO in the supernatants of the activated macrophages treated with FML were significantly higher than the activated macrophages treated with PBS (11.20 ± 1.80 μM/ml *vs* 7.84 ± 1.54 μM/ml, Tukey’s *post-hoc* test, *q* = 4.32, *P* = 0.013). As shown in Fig. 2, treatment of activated macrophages with FML (5 μg/ml) plus GL (at concentration of 10 and 20 μg/ml) significantly increased the NO production in comparison with FML treatment alone (11.20 ± 1.80 μM/ml *vs* 14.40 ± 1.90 μM/ml, Tukey’s *post-hoc* test, *q* = 4.12, *P* = 0.026 and 15.60 ± 1.66 μM/ml, Tukey’s *post-hoc* test, *q* = 5.67, *P* = 0.003) (Fig. 2).

**Fig. 3:**
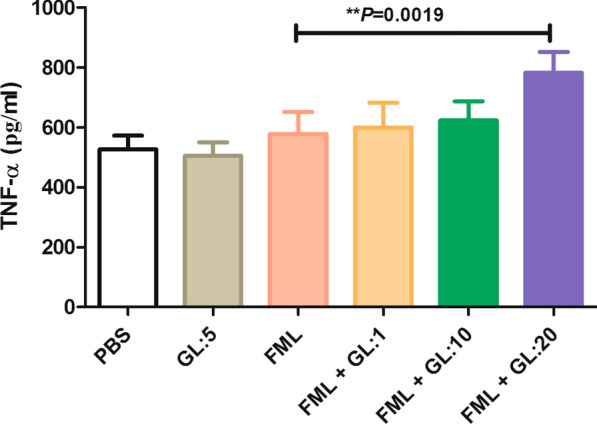
The effect of treatment with a combination of fucose-mannose ligand (FML) with glycyrrhizin (GL) on the production of TNF-α by activated macrophages. The production of TNF**-**α was significantly higher in the macrophages treated with the combination of FML and GL using a high concentration (20 μg/ml) compared to the activated macrophages treated with PBS, FML or GL alone (*P* = 0.0019). Results represent the mean (mean ± SD) of the three independent experiments with macrophages from seven mice per experiment (**P < 0.01). *Abbreviations*: PBS, phosphate-buffered saline

The measurement of TNF-α in the culture supernatant of activated macrophages, showed no significant differences between the FML-treated macrophages and PBS treated macrophages (577.6 ± 73.4 pg/ml *vs* 526 ± 45.6 pg/ml, Tukey’s *post-hoc* test, *q* = 1.75, *P* = 0.223). However, macrophages that were treated with FML in combination with 20 μg/ml GL showed a significant increase in the production of TNF-α in activated macrophages compared to the activated macrophages treated with FML alone (782.0 ± 69.49 *vs* 577.6 ± 73.4 pg/ml, Tukey’s *post-hoc* test, *q* = 7.00, *P* = 0.0019) (Fig. 3).

**Fig. 4:**
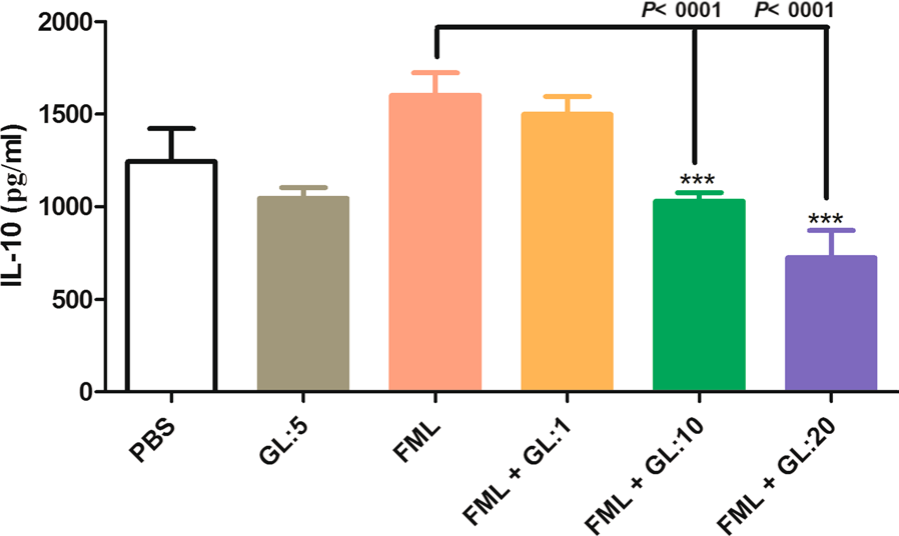
The effect of a combination of fucose-mannose ligand (FML) with glycyrrhizin (GL) on the production of IL-10 by activated macrophages. Treatment with FML together with GL at 10 and 20 μg/ml concentrations significantly reduced IL-10 production by activated macrophages compared to FML treatment alone. Results are expressed as the mean ± SD of triplicate cultures. Results represent the mean (mean ± SD) of three independent experiments with macrophages from seven mice per experiment (**P < 0.01). *Abbreviations*: PBS, phosphate-buffered saline

FML significantly increased IL-10 production from activated macrophages compared to the activated macrophages treated with PBS (1601 ± 54.11 pg/ml *vs* 1242 ± 79.68 pg/ml, Tukey’s *post-hoc* test, *q* = 6.80, *P* = 0.005). The concentration of IL-10 in the supernatant of the activated macrophages treated with a combination of FML and GL (at concentrations of 10 and 20 μg/ml) were significantly lower than the concentration of IL-10 in the supernatant of only FML-treated activated macrophages (1601 ± 54.11 pg/ml *vs* 1028 ± 46.2 pg/ml, Tukey’s *post-hoc* test, *q* = 10.87, *P* < 0.001, and 722.2 ± 147.8 pg/ml, Tukey’s *post-hoc* test, *q* = 16.68, *P* < 0.001) (Fig. 4).

**Fig. 5:**
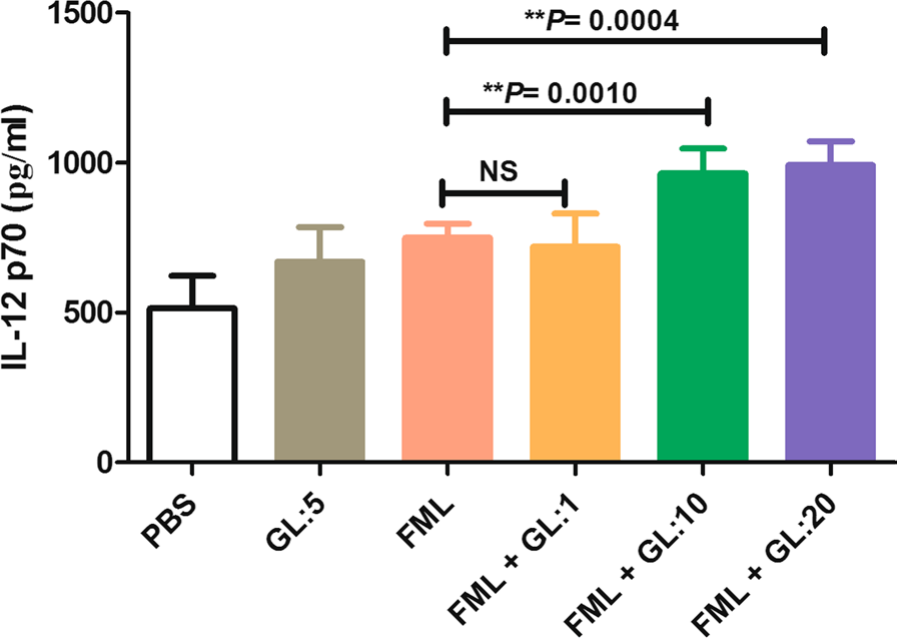
The effect of a combination of fucose-mannose ligand (FML) with glycyrrhizin (GL) on the production of IL-12p70 by macrophages. Treatment with FML and with 10 and 20 μg/ml concentrations of GL significantly increased IL-12p70 levels in the activated macrophages. Results are expressed as the mean ± SD of measurements from triplicate cultures (**P ≤ 0.001). *Abbreviations*: PBS, phosphate-buffered saline; ns, not significant

The results of our study showed that the co-treatment with FML and GL (at concentrations of 10 and 20 μg/ml) significantly increase the production of IL-12p70 from activated macrophages compared with the activated macrophages treated with FML alone (749.3 ± 47.5 pg/ml *vs* 991.6 ± 79.1 pg/ml, Tukey’s *post-hoc* test, *q* = 5.13, *P* < 0.001, and 964.6 ± 83 pg/ml, Tukey’s *post-hoc* test, *q* = 5.78, *P* < 0.0004) (Fig. 5). Also, there was a significant difference between the concentration of IL-12p70 produced from the activated macrophages treated with FML and the activated macrophages treated with PBS (749.3 ± 47.5 pg/ml *vs* 514.6 ± 48.37, Tukey’s *post-hoc* test, *q* = 5.60, *P* = 0.002) (Fig. 5).

## Discussion

Previous studies have shown that FML does not provide adequate immunogenicity and cannot efficiently stimulate the immune response of macrophages [9, 15]. GL is a well-known immunomodulatory component that stimulates immune response of infected macrophages [28]. Therefore, we assumed that treatment with a combination of FML and GL can improve the efficiency of macrophages against VL infection through the induction of protective cytokines and reactive nitrogen species (RNS). To evaluate this hypothesis, we studied the effects of FML in combination with GL on the production of TNF-α, IL-12p70, IL-10 and NO in murine peritoneal activated macrophages *in vitro*.

The results of our study indicated that the treatment of activated macrophages with FML plus GL leads to enhanced production of NO, TNF-α and IL-12p70 in comparison with FML treatment alone. Surprisingly, we found that co-treatment of macrophages with FML and GL markedly inhibits the production of IL-10 compared to FML treatment alone. The cytokine profile and NO levels in macrophages treated with a combination of FML and GL were similar to the patterns described in earlier studies on M1 macrophages [16].

NO has been demonstrated to be a principal effector molecule responsible for mediating the intracellular killing of *Leishmania* parasites, particularly the *L. donovani* complex [29]. The present study has shown that GL helps in enhancing the production of NO from FML-treated activated macrophages (Fig. 2). In accordance with the present results, previous studies have demonstrated that FML or GL alone enables an increase in NO production from activated macrophages [19, 24].

TNF-α plays a critical role in the control of intracellular pathogens, especially those that infect macrophages [30]. It has been demonstrated that TNF-α is required for the control of VL infection in humans through the stimulation of IFN-γ production [30]. Similarly, Tumang et al. [31] have also shown that endogenous TNF-α appears to be critical for both the initial acquisition of resistance to *L. donovani* and resolution of experimental VL infection. We know from our previous study that FML is unable to significantly enhance TNF-α levels in activated macrophages [19]. In the present study, we found that the treatment of activated macrophages with FML plus GL significantly increases the production of TNF-α, while treatment with FML or GL alone did not increase the production of TNF-α significantly. This outcome is contrary to that of Liu et al. [32] and Fu et al. [33] who found that GL inhibits the secretion of TNF-α by LPS-stimulated macrophages and mammary epithelial cells, respectively. It is difficult to explain these discrepancies, but it may be due to inhibition of LPS-induced nuclear factor-ĸB by GL, without suppression of nuclear factor-ĸB induced by MyD88-dependent downstream signaling [33].

VL is characterized by the absence of cytokines such as IFN-γ and IL-12 and cure of VL is associated with restoration of these cytokines [34]. Several reports have shown that IL-10 and IL-12 play opposite roles in control of VL infections [35]. IL-10 is associated with progression of VL, and IL-12 with the control of the disease caused by species of the *L. donovani* complex [35]. In a recent study, we demonstrated that FML increases the levels of both IL-12p70 and IL-10 in activated macrophages [19]. In the present study, we have shown that the use of FML in combination with GL exerts a strong booster effect on IL-12 and a suppressive effect on IL-10 production in activated macrophages. This finding is consistent with that of Dai et al. [23] who demonstrated the ability of GL to enhance LPS-induced IL-12 production in peritoneal macrophages. The results of the present study are also consistent with our earlier observations, which showed that GL significantly inhibits IL-10 secretion by LPS-stimulated RAW264.7 cells, a mouse macrophage cell line [32].

## Conclusions

Taken together, our findings suggest that GL can improve the immunostimulatory effect of FML on macrophages and lead to their polarization toward an M1-like phenotype, which is an efficient phenotype against *Leishmania* parasites. Further studies using animal models of VL are needed to evaluate the protective effect of the combined treatment with FML/GL.

## Data Availability

Data supporting the conclusions of this article are included within the article.

## Abbreviations

VL: visceral leishmaniasis
FML: fucose-mannose ligand
LPS: lipopolysaccharide
NO: nitric oxide
GL: glycyrrhizin
PBS: phosphate buffered saline
MTT: microwave theory and techniques
OD: optical density
